# Four new species of *Cymatodera* Gray from central and southern Mexico (Coleoptera, Cleridae, Tillinae)

**DOI:** 10.3897/zookeys.513.9935

**Published:** 2015-07-16

**Authors:** Alan F. Burke, Jacques Rifkind, Gregory Zolnerowich

**Affiliations:** 1 Department of Entomology, 123 Waters Hall, Kansas State University, Manhattan, KS 66506, USA; 2 Research Associate, California State Collection of Arthropods, 3294 Meadowview Road, Sacramento, CA 95832, USA

**Keywords:** Cleridae, *Cymatodera*, Mexico, brachyptery, Chiapas, Nevado de Jalisco, Sierra de Manantlan, Tehuacan

## Abstract

Four new species of *Cymatodera* are described from Mexico: *Cymatodera tortuosa* Burke & Rifkind, **sp. n.** from Hidalgo and Tamaulipas; *Cymatodera ortegae* Burke, **sp. n.** from Colima, Jalisco and Michoacan; *Cymatodera gerstmeieri* Burke & Rifkind, **sp. n.** from Chiapas; and *Cymatodera mixteca* Burke & Rifkind, **sp. n.** from Puebla and Guerrero. Male genitalia and other characters of taxonomic value are illustrated.

## Introduction

As part of an ongoing effort to catalog the diversity of Mexican Cleridae, the present work describes four new species of *Cymatodera* Gray from the central and southern states of Mexico. As previously discussed (Rifkind 2014, 2015), the diversity of the clerid genus *Cymatodera* in Mexico is extensive, but our knowledge of the group remains rudimentary. Many dozens of species await description and many more are likely to be discovered, particularly in areas distant from paved roads. Recent descriptions of species belonging to this genus include many endemics (Burke 2013; Burke and Zolnerowich 2014; Rifkind et al. 2010; Rifkind 2014, 2015), and here again, it is quite likely that the tally will increase as collecting efforts reach further into habitats such as relictual cloud forest and isolated mountain ranges that are known centers of endemism. Much attention has been focused on the rapid destruction or degradation of natural habitat in Mexico and other parts of Latin America, and the insufficiency of current resources dedicated to cataloguing disappearing biodiversity (Blackman et al. 2014; Armesto et al. 2007; Santibañez and Santibañez 2007; Trejo and Dirzo 2000). One positive trend is a recent increase in the number of trained taxonomists from these countries. Several workers in Mexico, for example, have undertaken faunistic and systematic studies of Cleridae with the result that the pace of description there is now higher than it has been in nearly a century (Burke 2013; Burke and Zolnerowich 2014; Rifkind et al. 2010, Toledo-Hernández et al. 2015). Should this trend continue, there may be more hope than previously thought for the assessment and conservation of critically threatened habitats in Mexico.

## Material and methods

Genitalia extraction and dissection procedures are similar to those outlined by Ekis (1977). Most of the morphological terminology used follows the works of Ekis (1977), Rifkind (1996) and Opitz (2010). Morphology of the male genitalia and pygidia are considered of primary importance as characters for the determination of new species in this descriptive work. Specimens were observed using a Leica MZ 7.5 stereomicroscope. Images were taken and measured using a Leica DFC 500 digital camera, and stacked using the software Zerene Stacker V. 1.04.

The following abbreviations are used in the description of the holotypes: TL = Total body length, HW = Maximum head width, HL = Head length, PW = Maximum pronotal width, PL = Pronotal length, EW = Maximum elytral width, EL = Elytral length.

Type material is deposited in the following collections:

CASC California Academy of Sciences Collection, San Francisco, California, USA

CIUM Colección de Insectos de la Universidad Autónoma del Estado de Morelos, Centro de Investigación en Biodiversidad y Conservación, UAEM, Mexico.

CNIN Colección Nacional de Insectos, Instituto de Biología, UNAM, DF, Mexico

EMEC Essig Museum of Entomology, University of California, Berkeley, USA

FMNH Field Museum of Natural History, Chicago, Illinois, USA

JEWC James E. Wappes Collection, San Antonio, TX, USA

KSUC Kansas State University Museum of Entomological and Prairie Arthropod Research Collection, Kansas State University, Manhattan, KS, USA

JNRC Jacques Rifkind Collection, Valley Village, CA, USA

RHTC Robert H. Turnbow Jr. Collection, Enterprise, AL, USA

SEMC University of Kansas, Snow Entomological Museum, Lawrence, KS, USA

TAMU Texas A&M Insect Collection, Texas A&M University, College Station, TX, USA

WFBM William F. Barr Entomological Museum, University of Idaho, Moscow, ID, USA

## Taxonomy

### Cymatodera tortuosa

Taxon classificationAnimaliaColeopteraCleridae

Burke & Rifkind sp. n.

http://zoobank.org/00F91AE7-8F5D-4A03-B70B-DD1B2BF851A0

**Figures 1–5.**
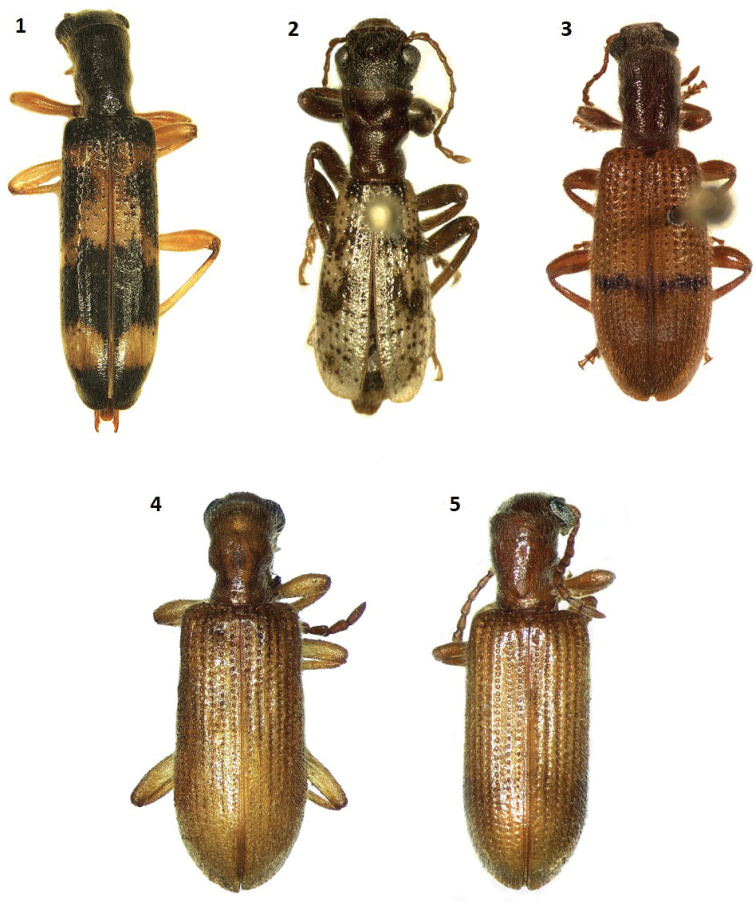
Habitus of: **1** *Cymatodera tortuosa* sp. n. (holotype male) **2** *Cymatodera ortegae* sp. n. (paratype male) **3** *Cymatodera gerstmeieri* sp. n. (holotype male) **4** *Cymatodera mixteca* sp. n. (holotype male) **5** *Cymatodera pallida* (male).

**Figures 6–10.**
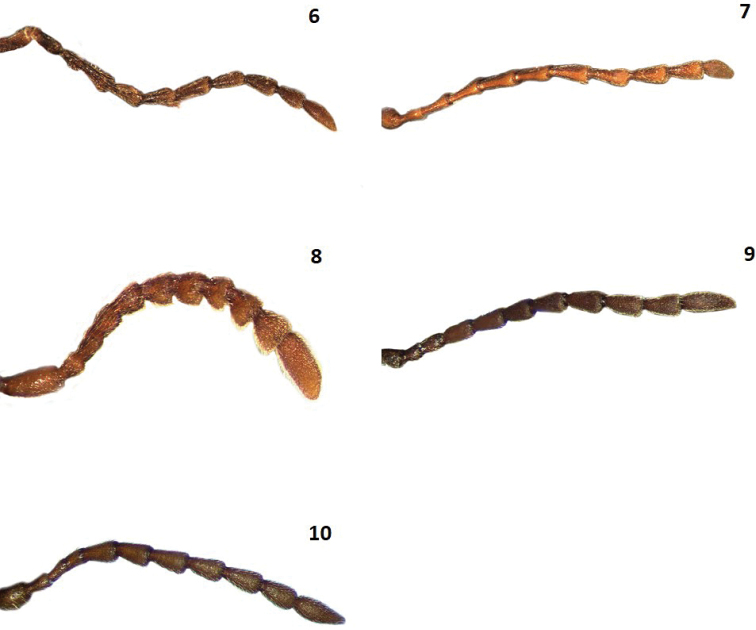
Antennae of: **6** *Cymatodera tortuosa* **7** *Cymatodera ortegae* **8** *Cymatodera gerstmeieri* **9** *Cymatodera mixteca* **10** *Cymatodera pallida*.

**Figures 11–22.**
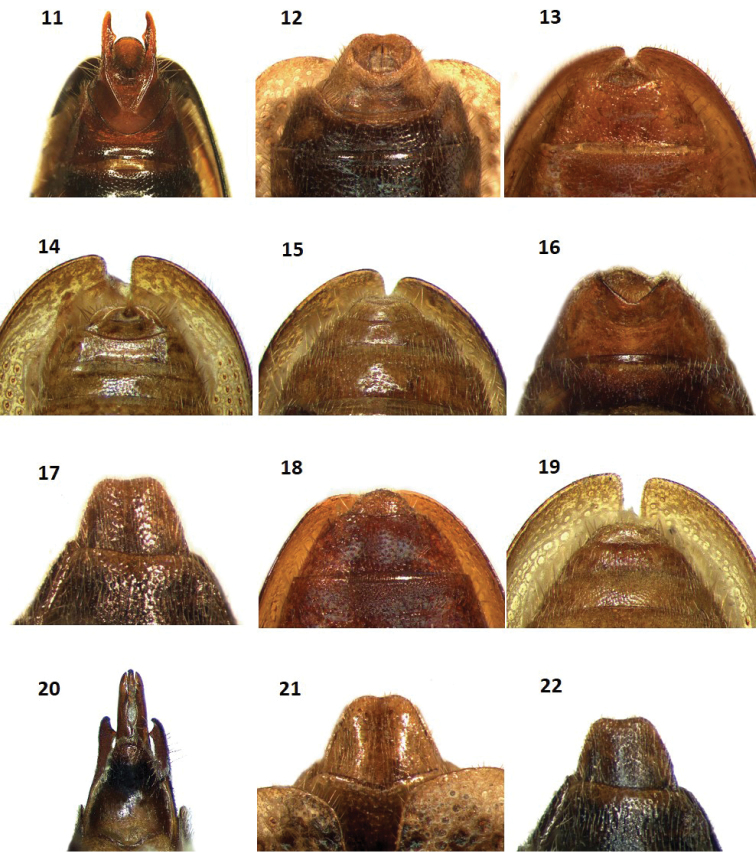
Pygidia of: **11** *Cymatodera tortuosa* (male, ventral view) **12** *Cymatodera ortegae* (male, ventral view) **13** *Cymatodera gerstmeieri* (male, ventral view) **14** *Cymatodera mixteca* (male, ventral view) **15** *Cymatodera pallida* (male, ventral view) **16** *Cymatodera tortuosa* (female, ventral view) **17** *Cymatodera ortegae* (female, ventral view) **18** *Cymatodera gerstmeieri* (female, ventral view) **19** *Cymatodera mixteca* (female, ventral view) **20** *Cymatodera tortuosa* (male, dorsal view) **21** *Cymatodera ortegae* (male, dorsal view) **22** *Cymatodera ortegae* (female dorsal view).

**Figures 23–26.**
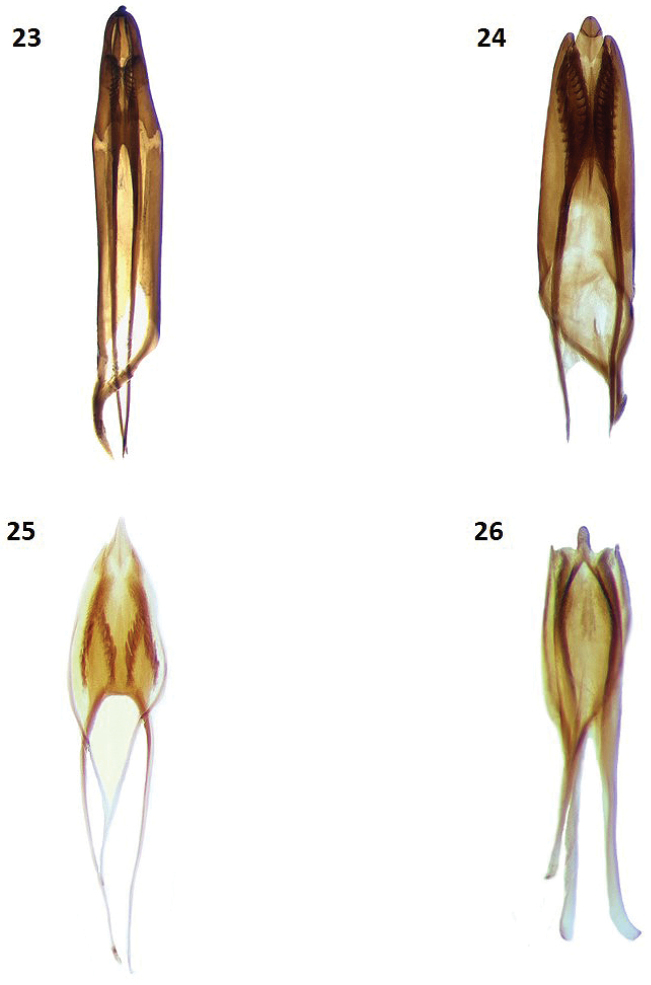
Male genitalia of: **23** *Cymatodera tortuosa* **24** *Cymatodera ortegae* **25** *Cymatodera gerstmeieri* **26** *Cymatodera mixteca*.

Figs 1 , 6 , 11 , 16 , 20 , 23

#### Type material

(n = 2). Holotype, red labeled, male: Mexico, Hidalgo, La Florida, municipio de Cardonal, Sitio 1A, 4-V-2014, S. Quiñonez; holotype deposited in CNIN. Paratype: 1 female: Mexico, Tamaulipas Mpio. Tula, La Presita, Canon de Coyote, 1,900 m, 16-III-1987, P. Kovarik, R. Jones, R. Trevino; paratype deposited in TAMU.

#### Differential diagnosis.

The new species can be separated from congeners by its unique combination of body form, antennal shape, size, color, and elytral fascia pattern. *Cymatodera tortuosa* superficially resembles a number of species that share a similar pattern of fuscous and testaceous elytral banding, such as *Cymatodera balteata* LeConte, *Cymatodera sirpata* Horn, *Cymatodera undulata* (Say), and *Cymatodera wolcotti* Barr. *Cymatodera tortuosa*, however, can be readily differentiated from those species based on clear differences in the male and female pygidium as well as discontinuity in geographic distribution. Specifically, the new species has the male pygidium distinctly modified (Fig. 11) compared to similar species; the female pygidium is moderately, V-shaped emarginate (Fig. 16), rather than broadly rounded posteriorly, as observed in similar species. As no specimens are known outside of central Mexico, it is possible that this new species is restricted to that region, whereas those listed previously are distributed for the most part in the south and southwestern United States, with only *Cymatodera balteata* ranging into the Mexican border states of Nuevo Leon and Tamaulipas.

#### Description.

Holotype male. Form elongate, slender; metathoracic wings present and fully developed. TL = 11.9 mm. Color: Head, pronotum and thorax piceous; elytra slightly lighter; antennae and mouthparts brunneous; legs testaceous; abdomen brunneous mesally, becoming pale testaceous laterally. Elytra with a pattern of testaceous markings and maculae as follows: the anterior 1/2 with a roughly X-shaped marking; each elytron at posterior 1/4 with a large, irregularly bordered, ovate macula narrowly separated at the elytral suture (Fig. 1).

Head: HL = 2.15 mm, HW = 1.95 mm. Measured across eyes wider than pronotum; surface smooth, moderately shiny, moderately, finely punctate; frons bi-impressed; sparsely clothed with short, very fine, recumbent setae, more profusely vested behind the eyes with longer setae; eyes moderately small, subsinuate, taller than wide, moderately emarginate in front, moderately protuberant laterally, separated by approximately 2.5 eye-widths. Antennae slender; loosely composed; extending beyond posterior margin of elytra; third antennomere about two times the length of second antennomere, fourth antennomere slightly longer than third antennomere; antennomeres 5–10 subequal in length; antennomeres 4–10 weakly serrate, serration very gradually increasing distally; last antennomere flattened apically, as long as tenth antennomere (Fig. 6).

Thorax: PL = 2.75 mm, PW = 1.9 mm. Pronotum elongate; widest at middle; middle slightly broader than anterior margin; sides constricted laterally; more strongly constricted behind middle; disc flat; moderately impressed in front of middle; subbasal tumescence feebly pronounced; surface rugulose; moderately, finely punctate; vested with short, fine, pale recumbent setae intermingled with less numerous, long erect setae, the latter more numerous laterally. Prosternum wider than long, rugulose, moderately puncticulate, scarcely vested with fine, pale, semirecumbent setae. Mesosternum smooth, shiny, feebly, coarsely, deeply punctate. Metasternum convex; rugose; moderately, shallowly punctate; moderately clothed with pale, semirecumbent setae, vestiture more abundant medially.

Legs: Femora clothed with short, recumbent setae interspersed with a few erect and semierect setae; tibiae moderately vested with some short and long erect and semierect setae; femora and tibiae transversely rugose.

Elytra: EL = 8.8 mm, EW = 2.6 mm. Anterior margin arcuately emarginate; wider than widest portion of pronotum; humeri pronounced; sides subparallel; widest on posterior fourth; disc convex; apex moderately dehiscent; elytral sculpturing as follows: anterior third set with regular, rather coarse and deep striae that abruptly diminish after anterior third and disappear entirely on posterior half; punctations at elytral base coarse and deep; surface moderately clothed with short, very fine, pale, recumbent setae intermixed with very few fine, long, erect setae.

Abdomen: Ventrites 1–5 rugulose, strongly convex; shallowly, moderately punctate; subequal in length; each ventrite with a pair of large, pale, shallow impressions near sides; surface clothed with fine, pale, moderately long, recumbent setae. Fifth ventrite (Fig. 11) moderately smooth; sides moderately oblique and arcuate; posterior margin broadly, deeply emarginate, emargination reaches medial portion of segment, posterolateral angles acuminate; sixth ventrite (Fig. 11) longer than wide, surface rugulose, with a pair of oblique, V-shaped, longitudinal carinae that initiate on anterior 1/4 and end slightly beyond segmental mid-length; highly modified distally, posterior margin deeply emarginate, emargination U-shaped, with the posterolateral angles each produced as a dorsally recurved and conspicuously elongate extension, the last third of each posterolateral extension bearing an internal spine or acuminate protuberance. Fifth tergite (Fig. 20) moderately convex; rugulose; lateral margins subparallel; posterior margin bisinuate, broadly, moderately deeply, triangularly emarginate at middle. Sixth tergite (Fig. 20) conspicuously longer than wide; subtriangular; surface strongly convex; rugulose; moderately, shallowly punctate; lateral margin oblique; posterior margin narrowly, moderately rounded, much surpassed by posterolateral extensions of the sixth ventrite.

Aedeagus 2.8 mm long; moderately robust; ratio of length of paramere to whole tegmen 0.45:1; tegmen fully covering phallus; parameres robust throughout their length; lateral margins feebly obtuse, subparallel, pointed distally; phallobase wide; phallic plate armed with a row of denticles along dorsal margin; phallobasic apodeme robust distally, moderately short; phallobasic struts rather slender throughout their length (Fig. 23).

Female: The female paratype is somewhat longer than the male, having a total body length of 13.8 mm. The female also differs from the male holotype by having the sixth ventrite strongly, deeply, V-shaped emarginate, lacking the pair of elongate, posterolateral extensions observed in the male (Figs 11, 20). Additionally, the female has a slightly paler integument than the male, with a less distinct elytral pattern anteriorly by comparison.

#### Distribution.

Presently known only from the northern portion of Hidalgo and the southeastern part of Tamaulipas, Mexico. The vegetation at the collecting localities is predominantly pine-oak forest (Fig. 29).

#### Etymology.

The specific epithet *tortuosa* (from the Latin *tortilis* or *tortuosus*, meaning twisted or winding), is a reference to the intricate and elaborate structure of the male pygidium of this species.

### Cymatodera ortegae

Taxon classificationAnimaliaColeopteraCleridae

Burke sp. n.

http://zoobank.org/27646149-9E6B-4397-ACD2-DB7E55F37A7C

Figs 2 , 7 , 12 , 17 , 21 , 22 , 24

#### Type material

(n = 30). Holotype red labeled, male: Jalisco, road to microondas Los Mazos, Sierra Manantlán, 1425-1610 m, 19°42'N, 104°24'W, 12 km SSD Autlan, mixed hardwood forest 15-VII-1993, R. L. Westcott; holotype deposited in CASC. Paratypes yellow labeled: 1 male: same data as holotype (WFBM); 1 female: Mexico: Jalisco, 81 km E of El Grullo, 6-X-1992, R. Turnbow (RHTC); 2 males, 1 female: Mex: Jalisco, N slope Nevado de Colima, 8000’, 17-VII-1990, J. Wappes (JEWC); 1 male, Jalisco, Autlán, Res. de la Biosfera Manantlán, Est. Cientifica Las Joyas, 19°35'443"N, 104°16'468"W, 30-VIII-2001, Col. V. H. Toledo (CIUM); 1 male, 2 females: Mexico, Jalisco, 2 km S La Manzanilla, 12-X-2001, F. Hovore (JNRC); 1 female: Mexico, Jalisco, Nevado de Colima, 8200’, Parque Nacional, 10.7 mi N Hwy 54, 17-IX-1986, [no collector data] (KSUC); 1 male: Mexico, Jalisco, 24.8 km SW Ciudad Guzman, 2286 m, 2-VII-1988, R. S. Anderson, pine-oak forest (JNRC); 1 male: Mexico: Sierra de Manantlan, Jalisco, Las Joyas, 1870 m, 18-VII-1985, J. Doyen, black and white light (EMEC); 1 male, 1 female: Mexico, Sierra de Manantlan, Jalisco, 1800-1900 m, 17-VII-1985, J. Doyen (EMEC); 4 females: Mexico, Jalisco, 5.4 km NE de Apango, 19 48 N, 103 41 W, 20-X-1996, beating dead leaf clumps of *Quercus* sp., R. L. Westcott (WFBM); 1 female: Mexico, Jalisco, Manantlan, Lab. Nat. Las Joyas, 8-VII-1988, F. A. Noguera and Y. A. Rodriguez (CNIC); 1 female: Mexico, Jalisco, 19 km E El Jazmin, (SW Ciudad Guzman), 2005 m, 19-VII-1993, pine-oak forest, R. L. Westcott, collected on *Quercus* sp. (WFBM); 2 males, 2 females: Mexico, Jalisco, km 3.5-4 Nevado de Colima, 24-VII-2011, R. Turnbow (RHTC); 1 female: Mex: Colima, nr El Terrero, 7800’, 18-VII-199, J. E. Wappes (JEWC); 1 male: Mexico, on elderberry stems, lot 72-11927, 27-VII-1972, Racine and Turk (WFBM); 1 male: Mexico, Colima, NW slope Nevado de Colima, 17-VII-1990, E. Giesbert (JNRC); 1 female: Colima, W rd. to El Terrero, 5000’, 3-5-X-1992, J. E. Wappes (JEWC); 1 male: Mexico, Michoacan, 2 km N Tancitaro, 2700-800 m, 26-I-1947, 53, T. H. Hubbell (JNRC).

#### Differential diagnosis.

The undulate fascia pattern on the elytral ground, the testaceous to slightly greenish integumental color, general body shape, and geographic distribution of the new species will, in combination, serve to separate it from other species of *Cymatodera*. *Cymatodera ortegae* appears to be allied to several Mexican congeners that share similar body shape, integumental color, brachypterous condition, and a reduced anterior elytral margin. Of these, *Cymatodera barri* Rifkind, *Cymatodera maculifera* Barr, and *Cymatodera monticola* Rifkind are most similar. Unlike *Cymatodera ortegae*, however, the males of *Cymatodera barri* and *Cymatodera maculifera* possess a distinct pair of feebly to moderately developed tubercles on the median posterior portion of the metasternum. Both sexes of these species lack the irregular, infuscate elytral pattern of the new species. *Cymatodera monticola* possesses distinctly different terminalia from *Cymatodera ortegae*, as well as sinuate elytral apices.

#### Description.

Holotype male. Medium sized, moderately slender anteriorly, rather robust posteriorly; brachypterous, TL = 13.1 mm. Color: Head, pronotum, thorax, femora and anterior portion of tibiae brunneous; posterior portion of femora and tarsomeres testaceous; antennae and mouthparts fuscous; abdomen fuscous, slightly darker than thorax, distal portion of abdominal segments with a depressed testaceous mark; elytral ground light testaceous, with a slight greenish tinge. Each elytron bearing a pair of irregular, sinuate, darkened fasciae: the first fascia located on anterior third, extending from the elytral suture to the epipleural fold, conspicuously slender proximal to elytral suture then abruptly widening before epipleural fold; the second fascia located at the elytral mid-length, moderately wide, extending from the elytral suture to before the epipleural fold. Punctation on elytral ground infuscate (Fig. 2).

Head: HL = 2.9 mm, HW = 2.5 mm. Large, measured across eyes wider than pronotum; surface rugose; frons bi-impressed; surface moderately punctate; clothed with short, fine, recumbent setae intermixed with long, semierect and erect setae; eyes moderately small, form subsinuate, longer than wide, moderately emarginate in front, very feebly bulging laterally, separated by approximately 3 eye-widths. Antennae slender; loosely composed; extending beyond posterior margin of elytra; antennomeres 2–3 subequal in length; fourth antennomere slightly longer than third; fifth antennomere very slightly longer than fourth, antennomeres 5–10 subequal in length, antennomeres 4–10 weakly serrate; last antennomere flattened apically, somewhat acuminate distally, approximately the same length as ninth antennomere (Fig. 7).

Thorax: PL = 4.05 mm, PW = 2.1 mm. Pronotum elongate; widest at middle; middle slightly broader than anterior margin; sides constricted subapically; more constricted behind middle; moderately impressed in front of middle; subbasal tumescence pronounced; surface rugose, moderately punctate, punctation rather coarse; clothed with short, pale, semirecumbent setae, intermingled with long, stiff, semierect pale setae. Prosternum convex; wider than long; surface rugose, shining, very feebly punctate. Mesosternum feebly convex; surface rugulose, moderately, deeply punctate, vested with fine, pale, recumbent setae. Metasternum convex; shortened longitudinally; surface rugulose, devoid of tubercles or carinae, moderately, shallowly punctate. Scutellum conspicuously wider than long; moderately setose.

Legs: Femora clothed with short, recumbent setae intermingled with less numerous erect and semierect setae; tibiae vested with short and long erect and semierect setae; femora and tibiae transversely rugose; metathoracic legs with tarsomeres longer than those of pro- and mesothoracic legs.

Elytra: EL = 7.7 mm, EW = 3.2 mm. Form: subovate (brachypterous type). Anterior margin arcuately emarginate; narrower than widest portion of pronotum; humeri very feebly indicated; sides widest on posterior fourth; disc convex; apex rounded, broadly dehiscent, not covering sixth ventrite; surface smooth, moderately clothed with short, fine, pale, recumbent setae intermixed with long, pale, fine, erect setae; sculpturing consisting of small, coarse punctures and larger punctation irregularly arranged from base to apex, punctures becoming less numerous behind anterior third, interstices about 3 × the diameter of punctures at elytral base.

Abdomen: Ventrites 1–5 rugulose; shallowly, moderately punctate; each segment with a pair of large, shallow impressions near sides; surface clothed with short, recumbent setae intermixed with less numerous, long, semi-erect setae. Fifth ventrite (Fig. 12) moderately convex; sides oblique, moderately arcuate; posterior margin broadly, very deeply emarginate. Sixth ventrite (Fig. 12) subquadrate; protruding laterally (visible in dorsal view); rugose; surface moderately concave; somewhat punctate; lateral margins oblique, feebly arcuate; posterior margin broadly, very deeply emarginate; posterolateral angles somewhat blunt, slightly procurved inwardly. Fifth tergite (Fig. 21) convex, rugulose; lateral margins slightly oblique; posterior margin narrowly, shallowly emarginate. Sixth tergite (Fig. 21) subquadrate; moderately convex; surface rugulose; lateral margins moderately oblique; posterolateral angles rounded; posterior margin broadly, shallowly, triangularly emarginate.

Aedeagus 2.3 mm long; ratio of length of parameres to whole tegmen 0.4:1; tegmen fully covering phallus; parameres subparallel, pointed at apex, lateral margins feebly oblique; phallobase moderately broad; phallus with copulatory piece feebly tapered distally; phallic plate armed with a row of moderately long denticles along the dorsal margin, these denticles increasing in size toward distal end; phallobasic apodeme short, robust, dilated distally; phallobasic struts slender throughout their length, each as long as phallobasic apodeme (Fig. 24).

Females of the type series differ from males by having the sixth ventrite (Fig. 17) with lateral margins moderately oblique, feebly arcuate, and posterior margin very feebly, shallowly, narrowly emarginate, rather than broadly, deeply, semicircularly emarginate, as observed in males (Fig. 12); additionally, females have the sixth tergite (Fig. 22) subquadrate, with the lateral margins oblique and the posterior margin very feebly, shallowly, narrowly emarginate. Females closely resemble males in other respects.

#### Variation.

Length of males range from 7.9–13.1 mm, females from 7.6–13.3 mm. Specimens examined have considerable variation in body size, and also in the shape of the fasciate pattern on the elytral ground, which ranges from almost incomplete and very narrow, to conspicuously wide, covering most part of the elytral ground. The color of these fasciae is also somewhat variable, ranging from dark testaceous to dark greenish. The elytral ground color ranges from light testaceous to light greenish.

#### Distribution.

Available specimens were collected in the central-occidental part of Mexico, in the states of Colima, Jalisco and Michoacan. Distribution appears limited to the southern tip of the Sierra Madre Occidental (Fig. 29).

#### Note.

This new species appears to be confined to mid-to-high altitude mountainous environments in the central-west portion of Mexico: specimens were collected on the slopes of Volcan Nevado de Colima in the states of Colima and Jalisco, and Cerro Tancitaro, in the northwestern region of the Mexican state of Michoacan. These mid and high altitude areas are dominated by pine and pine-oak forest stands.

#### Etymology.

The specific epithet is a patronymic honoring Cristina Ortega, a friend of the first author.

### Cymatodera gerstmeieri

Taxon classificationAnimaliaColeopteraCleridae

Burke & Rifkind sp. n.

http://zoobank.org/76E3C983-C185-4C7A-9BE2-1CF65B2858C7

**Figures 27–28.**
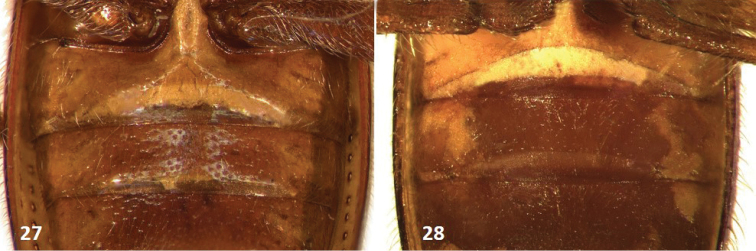
First and second ventrites of: **27** *Cymatodera gerstmeieri* (male) **28** *Cymatodera mitae* (male).

Figs 3 , 8 , 13 , 18 , 25 , 27

#### Type material

(n = 8): Holotype, red labeled, male: Mexico, Chiapas, El Aguacero, 680 m, 17-VI-1990, at light, R. A. Cunningham; holotype deposited in CSCA. Paratypes, yellow labeled: 4 males, 2 females: same data as holotype (JNRC), except 2 males and 1 female collected on 16-VI-1990, and 1 male collected on 01-IX-1990; 1 female: Mexico Chiapas, Aguacero, 16 km W Ocoz[ocuautla], 1-7-VII-1986, 2500’, J. E. Wappes (JEWC).

#### Differential diagnosis.

*Cymatodera gerstmeieri* is similar to a number of New World tilline species that share a testaceous to ferrugineous integument and a median, dark fascia on the elytral ground; those closest include *Cymatodera mitae* Burke, *Bogcia disjuncta* Barr, and *Cymatodera insignis* Schenkling. The new species can be separated from the former as follows: male specimens of *Cymatodera gerstmeieri* have the eleventh antennomere medially depressed, acuminate posteriorly, and approximately 2× longer than tenth antennomere (Fig. 8), while males of *Cymatodera mitae* have the eleventh antennomere cylindrical in shape, rounded posteriorly, and approximately 2.5–3× the length of tenth antennomere. Furthermore, males of *Cymatodera gerstmeieri* have a feebly developed longitudinal carina on the first ventrite, but lack a carina on the second ventrite (Fig. 27), whereas males of *Cymatodera mitae* have a well-developed longitudinal carina on the first ventrite, but also a somewhat less developed carina on the second (Fig. 28). The females of both species lack these carinae, but can be distinguished by the presence of a fuscous macula located on each elytral humeral angle in *Cymatodera mitae*, absent in *Cymatodera gerstmeieri*. The feebly to moderately serrate antennomeres 4-10 of *Cymatodera gerstmeieri* (Fig. 8) will easily separate it from *Bogcia disjuncta*, which has strongly serrate antennae. *Cymatodera gerstmeieri* somewhat recalls the Central American species *Cymatodera insignis*, with which it shares similar integumental color, antennal structure, and a median, slightly oblique, dark fascia. However, *Cymatodera insignis* bears a dark macula on the humeral angles, a longitudinal black macula at the posterolateral margin of the pronotum on either side, and has the posterolateral margins of the elytral ground narrowly darkened. These markings are absent in *Cymatodera gerstmeieri*.

#### Description.

Holotype male. Moderately small, rather robust, metathoracic wings complete, TL = 10.2 mm. Color: head, pronotum, prosternum, mesosternum ferrugineous-brown, the rest of the body uniformly testaceous brown. Each elytron with a fuscous, irregular fascia near the mid-length, extending from the elytral suture to the eighth row of striae, but not reaching the epipleural fold (Fig. 3).

Head: HL = 1.2 mm, HW = 1.85 mm. Measured across eyes wider than pronotum; surface feebly rugose, shiny; frons bi-impressed; moderately, coarsely punctate; vested with short, recumbent setae interspersed with some erect, stiff setae on and behind eyes; eyes rather rounded, moderately large, slightly longer than wide, feebly emarginate in front, bulging laterally. Antennae reaching posterior margin of pronotum; second antennomere 0.75× longer than third antennomere, antennomeres 3–10 subequal in length; antennomeres 2–3 subcylindrical; fourth antennomere feebly serrate; antennomeres 5–10 moderately serrate; last antennomere flattened apically, posterior margin acuminate, about 2× the length of tenth antennomere (Fig. 8).

Thorax: PL = 2.9 mm, PW = 1.5 mm. Pronotum rugose; moderately punctate; anterior margin as wide as middle; sides feebly constricted subapically; slightly more constricted behind middle; disc flat, very feebly impressed in front of middle; subbasal tumescence absent; surface clothed with moderately long, semirecumbent setae interspersed with some long, erect setae. Prosternum sparsely vested, feebly punctate. Mesosternum convex; surface shiny, moderately, shallowly punctate. Metasternum with surface rugulose, shiny, moderately, shallowly puncticulate.

Legs: Moderately vested with semierect and some recumbent setae; femora puncticulate, rugulose; tibia moderately punctate, rugulose.

Elytra: EL = 6.9 mm, EW = 4.4 mm. Broader than pronotum; humeri pronounced, rounded; sides subparallel, widest portion behind posterior third; disc flattened above; surface moderately rugulose; apices rounded, feebly dehiscent; elytral declivity gradual; integument clothed with short, semierect setae intermixed with fewer long, erect setae; surface bearing coarse punctation arranged in regular striae that gradually become smaller and shallower on posterior half, punctation not reaching elytral apex; interstices at elytral base about 1.2× the width of punctation.

Abdomen: Ventrites 1–5 rugulose, moderately, finely punctate, clothed with short, pale, fine, recumbent setae. First ventrite rather convex, subquadrate, conspicuously elevated at posterior 1/4 with a transverse, arcuate carina which does not attain posterolateral angles (Fig. 27). Fifth ventrite (Fig. 13) feebly convex; subquadrate in shape; surface shiny, moderately, shallowly, finely punctate; lateral margins oblique, somewhat arcuate; posterolateral angles rounded; posterior margin feebly, broadly emarginate. Sixth ventrite (Fig. 13) reduced; broader than long; subtriangular; lateral margins oblique, arcuate; hind margin moderately acuminate; surface feebly convex, shiny, moderately, finely, shallowly puncticulate. Fifth tergite subtriangular in shape, rugulose; lateral margin moderately oblique, posterior margin truncate. Sixth tergite subtriangular, broader than long; lateral margins strongly oblique, feebly arcuate; converging posteriorly; extending beyond apical margin of sixth ventrite.

Aedeagus 1.2 mm long; feebly sclerotized; wide; ratio of length of paramere to whole tegmen 0.45:1; tegmen partially covering phallus; parameres subtriangular; lateral margins obtuse, strongly oblique, pointed distally; phallobase wide; phallic plate armed with two long rows of moderately large denticles along dorsal and distal margins; phallobasic apodeme slender, somewhat shorter than endophallic struts; phallobasic struts slender throughout their length, 1.5 × the length of phallobasic apodeme (Fig. 25).

Females can be distinguished from males based on the structure of the pygidium. The sixth ventrite and the sixth tergite are broadly rounded posteriorly in females (Fig. 18). Other characters are constant in both sexes.

#### Variation.

Length of males ranges from 8.7–10.2 mm; length of females from 9.5–13.1 mm. The elytral fascia is slightly variable in width, extending from the elytral suture to the epipleural fold in one male and one female, but incomplete in remaining individuals. Two males and one female in the type series have slightly paler elytra than the male holotype.

#### Distribution.

**Figure 29.**
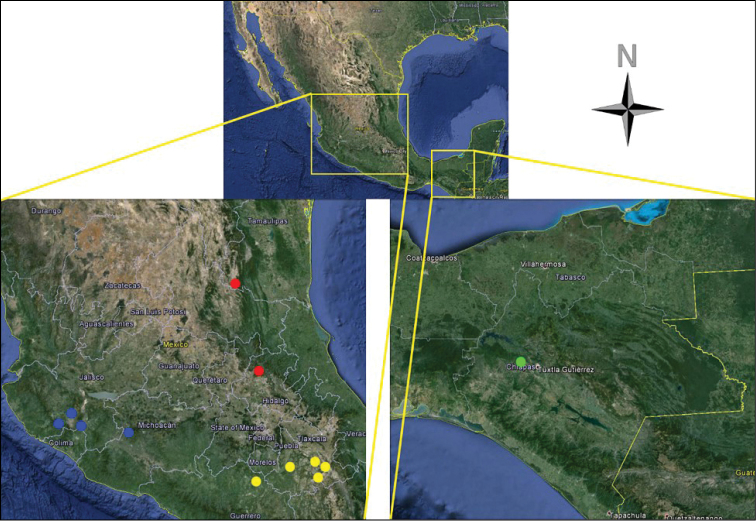
Map of central and south Mexico showing geographic position of collecting localities for: *Cymatodera tortuosa* (red circles); *Cymatodera gerstmeieri* (green circle); *Cymatodera mixteca* (yellow circles); and *Cymatodera ortegae* (blue circles).

All specimens in the type series were collected in the locality of El Aguacero, approximately 10 miles northwest of Ocozocuautla, Chiapas, Mexico. The elevation at the type locality is approximately 650 m and the vegetation is predominantly tropical deciduous forest (Fig. 29).

#### Etymology.

We name this beetle for Prof. Dr. Roland Gerstmeier (Technische Universität München, Germany), in recognition of his many contributions to the study of Cleridae.

### Cymatodera mixteca

Taxon classificationAnimaliaColeopteraCleridae

Burke & Rifkind sp. n.

http://zoobank.org/580DD2DD-2760-4140-9FCC-5D1E2B495D65

Figs 4 , 9 , 14 , 19 , 26

#### Type material

(n = 16): Holotype, red labeled, male: Cacaloapan, Puebla, Mexico, 26-IV-1962, L. A. Stange. Holotype deposited in CASC. Paratypes, yellow labeled: 1 male, 1 female: same data as holotype (FMNH); 2 males: Mexico, Puebla, 2 mi SW Tehuacan, 5300’, 4-X-1975, blacklight trap, 2300-0600, Powell (EMEC); 1 male: Tehuacan, Puebla, Mexico, 23-VI-1953, P. D. Hurd (JNRC); 1 female: 82 km NE Tehuacan, Puebla, Mexico, 5480 ft, rt. 2A, km 242, 7-VI-1948, desert, at light, F. Werner and W. Nutting (KSUC); 2 males, 1 female: Mexico, Puebla, 10 km N Tehuacan, 1650 m, 20-VII-1987, J. T. Doyen (EMEC); 1 female: Mexico, Puebla, 5 mi SW Zapotitlan, 8-VII-1973, Mastro and Schaffner (TAMU); 1 female: Mexico, Puebla, 8 mi SE Tehuitzingo, 29-VI-1961, 4100’, University of Kansas, Mexico expedition (SEMC); 1 male: Mexico, Puebla, 6 mi SW Tehuacan, 7-VII-1973, taken at light, Mastro and Schaffner (TAMU); 3 females: Mexico, Guerrero, Mexcala, 29-VI-1959 P. D. Hurd (EMEC);

#### Differential diagnosis.

*Cymatodera mixteca* is most similar to the allopatric *Cymatodera pallida* Schaeffer, but the two species can be readily differentiated based on the structure of the antennae. Antennomeres 2–3 of *Cymatodera mixteca* are about the same length and width but shorter and narrower than the fourth antennomere (Fig. 9), while antennomeres 2–4 are about the same length and width but shorter and narrower than the fifth antennomere in *Cymatodera pallida* (Fig. 10). The elytral integument in *Cymatodera mixteca* is uniformly pale-testaceous to testaceous (Fig. 4) while *Cymatodera pallida* has a faint, wide, transversal, dark-testaceous band on the last third of the elytral ground (Fig. 5). In addition, *Cymatodera mixteca* is restricted to central Mexico while *Cymatodera pallida* is found in the southwest portion of the United States and the northern state of Chihuahua, Mexico. Male pygidia of *Cymatodera mixteca* and *Cymatodera pallida* (Figs 14–15) closely resemble one another and will not serve to separate these species. The similar and possibly sympatric species *Cymatodera cylindricollis* Chevrolat is darker and moderately larger than *Cymatodera mixteca*.

#### Description.

Holotype male. Small, moderately slender, metathoracic wings complete. TL = 9.3 mm. Color: head, pronotum, prosternum, mesosternum and mouthparts testaceous; remainder of body pale testaceous (Fig. 4).

Head. HL = 1.1 mm, HW = 1.6 mm. Measured across eyes wider than pronotum; surface feebly rugose, shiny; frons not bi-impressed; moderately, finely punctate; vested with pale, short, recumbent, fine setae interspersed with some erect, fine, long and less numerous setae; eyes moderately rounded, large, slightly longer than wide, feebly emarginate in front, conspicuously bulging laterally. Antennae long, extending to posterior half of elytral length; second and third antennomere small, slender, about the same length; fourth antennomere about 3× longer than third antennomere, antennomeres 4–10 robust, moderately elongate, subequal in length; antennomeres 2–3 subcylindrical; antennomeres 4–10 moderately serrate; last antennomere acuminate posteriorly, flattened apically, about the same length of tenth antennomere (Fig. 9).

Thorax: PL = 1.7 mm, PW = 0.9 mm. Pronotum moderately rugose, feebly, finely punctate; anterior margin as wide as middle and posterior margin; sides feebly constricted subapically; more constricted behind middle; disc flat, feebly impressed in front of middle; anterior pronotal impression present, subbasal tumescence obvious; surface moderately clothed with pale, stiff, short and long, semirecumbent setae. Prosternum sparsely vested, feebly, finely punctate. Mesosternum convex; surface shiny, smooth, moderately, shallowly punctate. Metasternum with surface feebly rugose, moderately, shallowly puncticulate. Scutellum ovoid, wider than long, posteriorly emarginate.

Legs: Moderately vested with pale, fine, recumbent setae intermixed with some scattered, very long, pale, stiff setae; femora transversally rugulose; tibia feebly punctate, longitudinally, finely rugulose.

Elytra: EL = 4.9 mm, EW = 2.1 mm. Broader than pronotum; humeri pronounced, rounded; sides slightly ovoid; widest portion at posterior fourth; disc moderately flattened above, slightly depressed medially, smooth; apices subtriangular, feebly dehiscent; elytral declivity steep; integument clothed with short, pale, fine, recumbent setae intermixed with long, erect, pale, stiff setae; sculpture consisting of moderately coarse punctation arranged in regular striae that gradually become smaller and shallower on toward apex, punctation disappear before elytral apex; interstices at elytral base smooth, about 2.0× the width of punctation.

Abdomen: Ventrites 1–4 shiny, smooth; feebly, finely punctate; clothed with few short, pale, fine, recumbent setae; posterior margins truncate; lateral margins not depressed. Fifth ventrite (Fig. 14) conspicuously wider than long; surface smooth, shiny, moderately concave; lateral margins oblique, finely arcuate; posterior margin broadly, shallowly emarginate. Sixth ventrite (Fig. 14) small; broader than long; subtriangular; surface shiny, smooth, very finely rugulose, medially convex; lateral margins strongly oblique, arcuate, hind margin broadly, very shallowly emarginate; posterolateral angles rounded. Fifth tergite subquadrate; rugulose; lateral margin moderately oblique, posterior margin truncate. Sixth tergite subquadrate, broader than long; surface concave; lateral margins oblique, feebly arcuate; posterior margin truncate; posterolateral angles broadly rounded. Sixth tergite extending slightly beyond the apical margin of sixth ventrite.

Aedeagus 0.9 mm long; feebly sclerotized; moderately wide; ratio of length of paramere to whole tegmen 0.3:1; tegmen partially covering phallus; parameres ovoid; lateral margins obtuse, oblique, pointed distally; phallobase wide; phallic plate devoid of denticles, distal portion of phallic plate spinous, spines reduced; phallus rounded at apex, conspicuously wide at middle; phallobasic apodeme robust, swollen distally, longer than phallobasic struts; phallobasic struts moderately robust, swollen distally, approximately 1.2 × the length of phallobasic apodeme (Fig. 26).

Females of the type series can be differentiated from males by the shape of the sixth ventrite. This segment is broadly rounded posteriorly (Fig. 19), rather than shallowly emarginate, as in males (Fig. 14). Remaining characters are constant in both sexes.

#### Variation.

Length of males ranges from 6.3–8.4 mm; length of females from 7.1–7.9 mm. Individuals in the type series vary somewhat in integument color, ranging from pale testaceous to brown. Such color variation is observable in male and female members in the type series. Remaining characters in the type series remain consistent.

#### Distribution.

The type series was collected from various localities in the Sierra Mixteca of Mexico, specifically in the south and southwestern portion of the state of Puebla, and in central Guerrero state (Fig. 29). This region is characterized by tropical deciduous to thorny forest habitats.

#### Etymology.

The specific epithet makes allusion to the regional home of the Mixteca people, and of this new species.

## Supplementary Material

XML Treatment for Cymatodera tortuosa

XML Treatment for Cymatodera ortegae

XML Treatment for Cymatodera gerstmeieri

XML Treatment for Cymatodera mixteca
